# Dynamic changes in morphology, gene expression and microbiome in the jejunum of compensatory‐growth rats induced by protein restriction

**DOI:** 10.1111/1751-7915.13266

**Published:** 2018-04-06

**Authors:** Yizhi Zhu, Chao Shi, Qingyan Niu, Jing Wang, Weiyun Zhu

**Affiliations:** ^1^ Jiangsu Key Laboratory of Gastrointestinal Nutrition and Animal Health Laboratory of Gastrointestinal Microbiology College of Animal Science and Technology Nanjing Agricultural University Nanjing 210095 China

## Abstract

We previously reported that protein‐restricted rats experienced compensatory growth when they were switched to a normal protein diet (NPD). This study aimed to investigate the changes in gene expression and microbiome in the jejunum of compensatory‐growth rats. Weaned Sprague‐Dawley rats were assigned to an N group, an LN group and an L group. The rats in the L and N groups were fed a low protein diet (LPD) and the NPD respectively. The rats in the LN group were fed with the LPD for 2 weeks, followed by the NPD. The experiment lasted 70 days, and the rats were sacrificed for sampling on days 14, 28 and 70 to determine the jejunal morphology, microbiome and gene expression related to digestive, absorptive and barrier function. The results showed that, although rats in the LN group had temporarily impaired morphology and gene expression in the jejunum on day 14 in response to the LPD, they had improved jejunal morphology and gene expression related to jejunal function on day 28 compared to rats in the N group. This improvement might promote compensatory growth of rats. However, lower expression of genes related to nutrient absorption and undifferentiated villous height (VH) were observed in the jejunum of rats in the LN group on day 70. In contrast, rats in the L group had lower VH on day 28 and day 70, while the expression of absorptive genes increased on day 28 compared to rats in the N group. Additionally, dramatic microbial changes in the jejunum of compensatory‐growth rats were observed, principally for *Lactobacillus*,* Streptococcus*,* Corynebacterium* and *Staphylococcus*. Moreover, the abundance of *Lactobacillus*,* Streptococcus*,* Corynebacterium* and *Staphylococcus* significantly correlated with gene expression in the jejunum as revealed by the correlation analysis.

## Introduction

The small intestinal tract is crucial for host, as it provides nearly the whole nutrient requirement and regulates host endocrinology and immunity (Borgstrom *et al*., 1979; Murphy and Bloom, 2006; Peterson and Artis, 2014). Particularly, jejunum is a site where violent interactions between mucosal cells, microbiota and nutrients occur, establishing the homoeostasis of host metabolism (Aidy *et al*., 2013). However, jejunum harbours a relatively fewer microbes compared to the large intestine, due to the harsh environment for microbial life, such as short transit time, excretion of gastric acid, digestive enzymes and bile acids (Zoetendal *et al*., 2012). Thus, the effect of microbiota in the proximal parts of small intestine on host metabolism, such as jejunum, has remained largely unexplored to date. Aidy *et al*. (2013) reported that germ‐free mice transplanted with faecal microbiota exhibited modified gene transcripts and a metabolic shift from an oxidative energy supply to anabolic metabolism in the jejunal mucosa, indicating the importance of jejunal microbiota in regulating host gene expression and metabolism. Hence, jejunum together with the residing microbiota plays an important role in host metabolism.

We previously demonstrated that transiently protein‐restricted rats experienced compensatory growth after they were switched to the NPD. A higher feed conversion rate of rats during the compensatory‐growth period was observed, and the compensatory growth of rats was accompanied by dramatic changes in microbial composition and metabolism in colon, with increased production of short chain fatty acids (SCFAs) (Zhu *et al*., 2017). However, the mechanism behind the high feed conversion rate and rapid growth remains unknown. We speculate that the microbial composition in jejunum of rats also changed during compensatory growth, which further affected gene expression in jejunal mucosa to promote nutrient digestion and absorption. The purpose of this study is to investigate the dynamic changes in morphology, gene expression and microbiome in the jejunum of compensatory‐growth rats.

## Results

### Serum hormones

**Figure 1:**
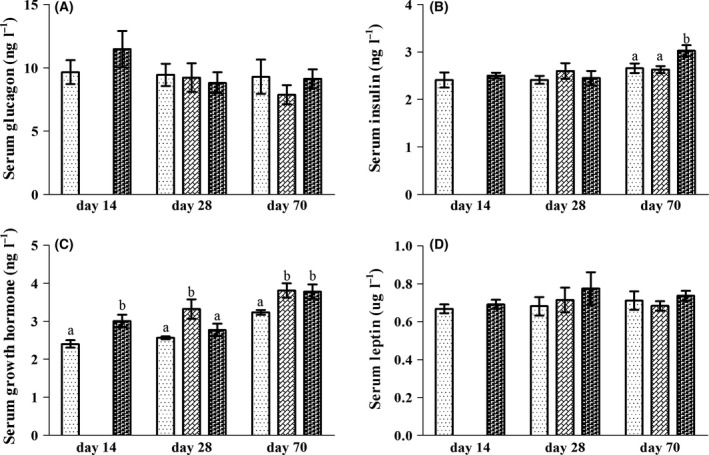
The serum concentrations of glucagon (**A**), insulin (**B**), growth hormone (**C**) and leptin (**D**) of rats in L (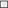), LN (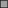) and N (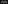) groups on day 14, day 28 and day 70. Values are presented as the mean ± SEM. Different letters among groups on the same day indicate a significant difference, *P *<* *0.05.

To achieve the experimental purpose, an N group, an L group and an LN group were involved in this study. The rats in the N group were fed with a normal protein diet throughout the experiment as a control group, while the rats in the L group were fed with a low protein diet consistently. On day 14, 12 rats in the L group were randomly selected, reassigned to six cages in average and were fed with the NPD afterwards, thus being the LN group. Throughout the experiment, no differences were found in the concentrations of serum glucagon and leptin of rats among L, LN and N groups (Fig. 1A and D). The serum insulin concentration of rats in all groups showed no differences on day 14 and day 28 (Fig. 1B). However, the concentration of insulin decreased in the L and LN groups compared to the N group on day 70. Insulin concentrations in the L and LN groups did not differ. The serum growth hormone (GH) concentration in the L group decreased on day 14 and day 70, but remained unaffected on day 28 compared to the N group (Fig. 1C). However, the GH concentration in the LN group was higher than in the L and N groups on day 28. On day 70, the GH in the LN group remained higher than in the L group and did not differ from the N group.

### Jejunal morphology

Morphological analysis revealed that the villous height (VH) in the jejunum of rats in the L group was unaffected on day 14, but decreased in the L group on day 28 and day 70 compared to the N group (Table 1). However, the VH in the jejunum of rats in the LN group was higher than for the rats in the L and N groups on day 28. On day 70, the VH in the LN group did not differ from the N group but was higher than in the L group. No difference was observed in the crypt depth (CD) and VH/CD ratio among all groups on day 14, day 28 and day 70.

**Table 1:** The villous height (VH), crypt depth (CD) and villous height/crypt depth ratio (VH/CD) in the jejunum of rats in each group

Item	L	LN	N	SEM	*P* value^a^
Day 14					
VH	519.42	–	512.04	27.68	0.90
CD	107.60	–	128.38	5.61	0.06
VH/CD ratio	4.90	–	4.05	0.32	0.19
Day 28					
VH	465.35^a^	590.65^b^	527.46^c^	14.78	0.00
CD	124.37	144.15	133.56	6.31	0.47
VH/CD ratio	3.89	4.08	4.12	0.18	0.87
Day 70					
VH	574.56^a^	653.75^b^	664.80^b^	12.75	0.00
CD	159.82	136.08	144.31	8.04	0.50
VH/CD ratio	3.91	5.10	4.68	0.28	0.22

Means in a same row with different superscripts indicate a significant difference (*n *=* *6, *P *<* *0.05).

a *P* value is the significance of independent samples *t*‐test or the significance of ANOVA.

### Gene expression in the jejunum

**Figure 2:**
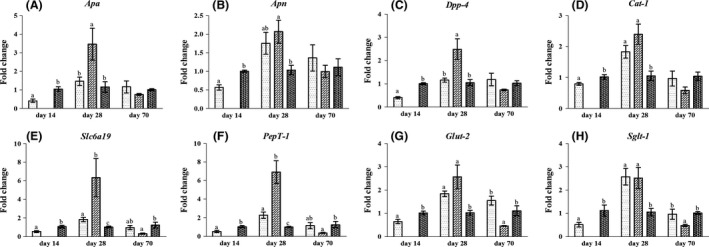
The expression of genes related to nutrient digestion (A–C) and absorption (D–H) in the jejunal mucosa of rats in the L (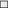), LN (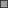) and N (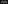) groups on day 14, day 28 and day 70. Values are presented as the mean ± SEM. Different letters among groups on the same day indicate a significant difference, *P *<* *0.05.

The expression of peptidase‐digesting genes, including aminopeptidase A (*Apa*), aminopeptidase N (*Apn*) and dipeptidyl peptidase 4 (*Dpp‐4*), was significantly downregulated in the jejunum of rats in the L group on day 14 compared to the N group (Fig. 2A–C). However, the expression of *Apa*,* Apn* and *Dpp‐4* was higher in the jejunum of rats in the LN group on day 28 compared to the N group, but remained unaffected on day 70. Moreover, the expression of *Apa* and *Dpp‐4* in the LN group was higher than in the L group on day 28, and no difference was observed in the expression of *Apa*,* Apn* and *Dpp‐4* between the L and LN groups on day 70. In contrast, there was a trend in the increase in the expression of *Apn* in the L group on day 28 compared to the N group (*P* < 0.1). The expression of *Apn* on day 70 and the expression of *Apa* and *Dpp‐4* on day 28 and day 70 in the L group were not affected compared to the N group.

The expression of nutrient‐absorbing genes, including cationic amino acid transporter 1 (*Cat‐1*), solute carrier family 6 member 19 (*Slc6a19*), peptide transporter 1 (*PepT‐1*), glucose transporter 2 (*Glut‐2*) and sodium‐dependent glucose transporter 1 (*Sglt‐1*), decreased in the jejunum of rats in the L group on day 14 compared to the N group (Fig. 2D–H). However, the rats in the LN group showed higher expression of *Cat‐1*,* Slc6a19*,* PepT‐1*,* Sglt‐1* and *Glut‐2* on day 28 in the jejunum compared to the N group but had significantly decreased expression of *Slc6a19*,* PepT‐1*,* Glut‐2* and *Sglt‐1* in the jejunum on day 70. Moreover, the expression of *Slc6a19* and *PepT‐1* in the LN group was higher than in the L group on day 28, and the expression of *Glut‐2* and *Sglt‐1* in the LN group was lower than that in the L group on day 70. In contrast, the expression of *Slc6a19*,* PepT‐1*,* Glut‐2* and *Sglt‐1* was upregulated and *Cat‐1* tended to be augmented (*P* < 0.1) in the L group on day 28 compared to the N group. All genes in the L group remained unaffected on day 70 compared to the N group.

**Figure 3:**
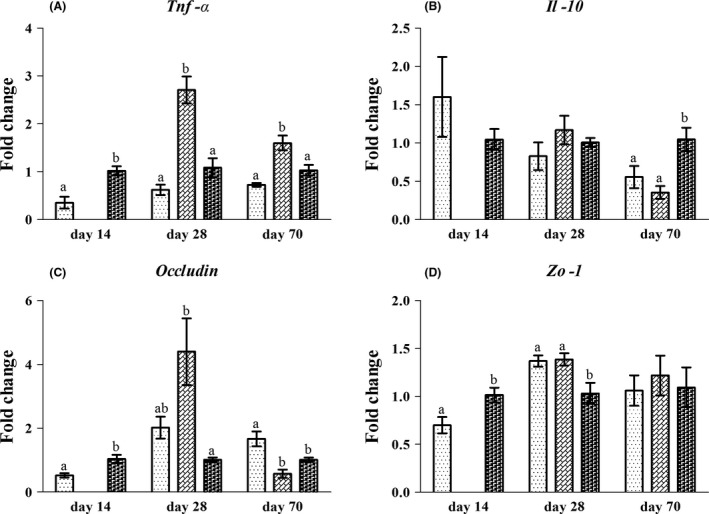
The gene expression of inflammatory cytokines (**A**–**B**) and tight junction proteins (**C**–**D**) in the jejunal mucosa of rats in the L (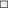), LN (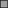) and N (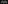) groups on day 14, day 28 and day 70. Values are presented as the mean ± SEM (*n *=* *6). Different letters among groups on the same day indicate a significant difference, *P *<* *0.05.

To reveal the innate immune function and barrier function in the jejunum, we assayed the expression of several selected cytokines and tight junction proteins. The results (Fig. 3A) showed that the expression of tumour necrosis factor‐α (*Tnf‐α*) in the jejunum of rats in the LN group was higher than for the rats in the L and N group on day 28 and day 70. In contrast, the expression of *Tnf‐α* decreased in the jejunum of rats in the L group on day 14 and tended to be inhibited on day 70 (*P* < 0.1) compared to the N group. The expression of the anti‐inflammatory cytokine interleukin‐10 (*Il‐10*) had no significant difference between the L and N groups on day 14 and among the L, LN and N groups on day 28. However, the expression of *Il‐10* was significantly decreased in the jejunum of rats in the L and LN groups on day 70 compared to the N group (Fig. 3B). No difference was detected in the expression of *Il‐10* between the L and LN groups. For gene expression of tight junction proteins (Fig. 3C and D), the expression of *Occludin* and *Zo‐1* was decreased in the jejunum of rats in the L group on day 14 compared to the N group. However, the expression of *Occludin* and *Zo‐1* was increased in the jejunum of rats in the LN group on day 28; the expression of *Occludin* tended to decrease in the LN group on day 70 (*P* < 0.1), and *Zo‐1* remained unaffected. Moreover, the expression of *Occludin* in the LN group was lower than in the L group on day 70. No difference was observed in the expression of *Occludin* on day 28 and the expression of *Zo‐1* on day 28 and day 70 between the L and LN groups. In contrast, the expression of *Occludin* in the jejunum of rats in the L group on day 28 tended to increase (*P* < 0.1) and significantly increased on day 70. In addition, the expression of *Zo‐1* increased in the L group on day 28, but remained unaffected on day 70 compared to the N group.

### Microbial community in the jejunal content

In this study, the average number of partial 16S rRNA gene reads detected was 51289, with 50632 valid sequences (Table S3). The overall number of operational taxonomic units (OTUs) detected was 440, based on a 97% sequence similarity between reads (Fig. S1). The sampling was sufficient to evaluate the bacterial community profile as indicated by the rarefaction curves (Fig. S2). The Shannon and Simpson indexes showed that the bacterial diversity in the jejunum of rats in the L group was not affected on day 14, day 28 and day 70 compared to the N group (Fig. S3). Moreover, the bacterial diversity in the LN group did not differ from the N group on day 28. However, it significantly increased in the LN group on day 70 compared to the N group. Moreover, the bacterial diversity in the LN group on day 70 was higher than in the L group on day 70, as indicated by the Simpson index.

**Figure 4:**
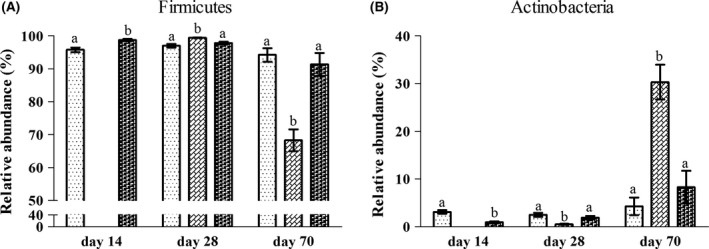
The altered abundance of bacteria in the jejunal content of rats at the phylum level (A–B) in the L (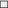), LN (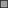) and N (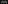) groups on day 14, day 28 and day 70. Values are presented as the mean ± SEM (*n *=* *6). Different letters among groups on the same day indicate a significant difference, *P *<* *0.05.

The bacterial composition was assessed at different taxonomic levels. At the phylum level, the dominant bacterial groups were Firmicutes, Actinobacteria, and Proteobacteria, accounting for 99.78% of the total sequences on average (Fig. S4). The rarely detected phyla, including Cyanobacteria/Chloroplast, Fusobacteria, Candidatus Saccharibacteria and Bacteroidetes, which had an average relative abundance <0.1%, were not involved in the subsequent statistical analysis. Statistically, at the phylum level, the abundance of Firmicutes and Actinobacteria in the jejunum significantly changed among the groups on day 14, day 28 and day 70 (Fig. 4A and B). The abundance of Firmicutes in the jejunum of rats in the L group was higher than in the N group on day 14, but it was not affected on day 28 and day 70. In addition, the abundance of Actinobacteria in the L group was significantly higher than the N group on day 14 and did not significantly differ from the N group on day 28 and day 70. In response to the diet switch from the LPD to the NPD, a significantly higher abundance of Firmicutes on day 28 and significantly lower abundance of Firmicutes on day 70 were observed in the jejunum of rats in the LN group compared to the L and N groups. However, the abundance of Actinobacteria in the jejunum of rats in the LN group was significantly lower than in the L and N groups on day 28 and was significantly higher than in the L and N groups on day 70. These results indicate that the relative abundance of Firmicutes and Actinobacteria in the jejunum actively changed in response to dietary treatments.

**Figure 5:**
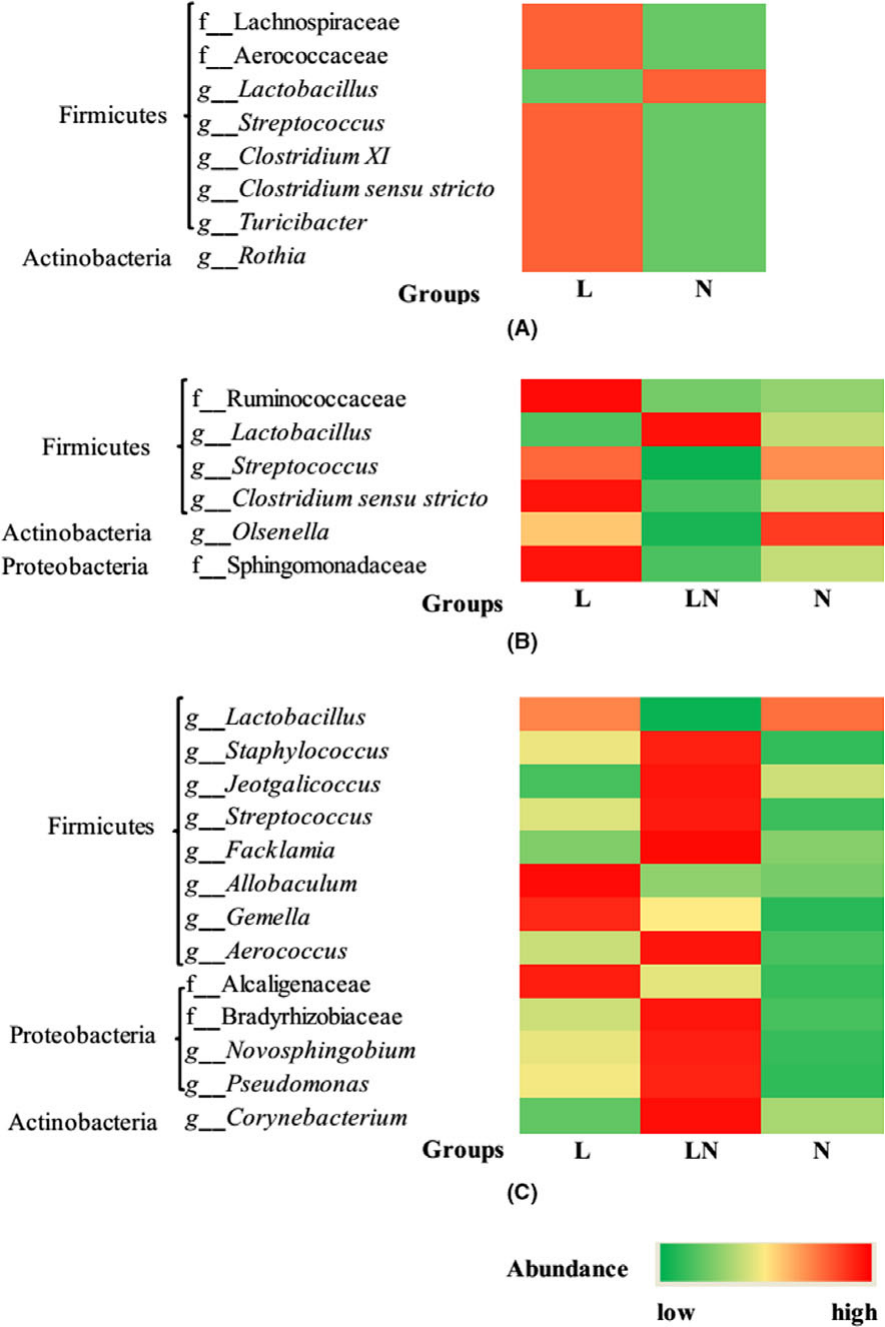
The altered bacterial groups among the top 20 predominant bacterial taxa in the jejunal content at the family and genus level on day 14 (**A**), day 28 (**B**) and day 70 (**C**). The intensity of colours represents the abundance of bacterial groups. The figure also clearly shows which phylum the altered bacterial groups at the family and genus level belongs to.

**Figure 6:**
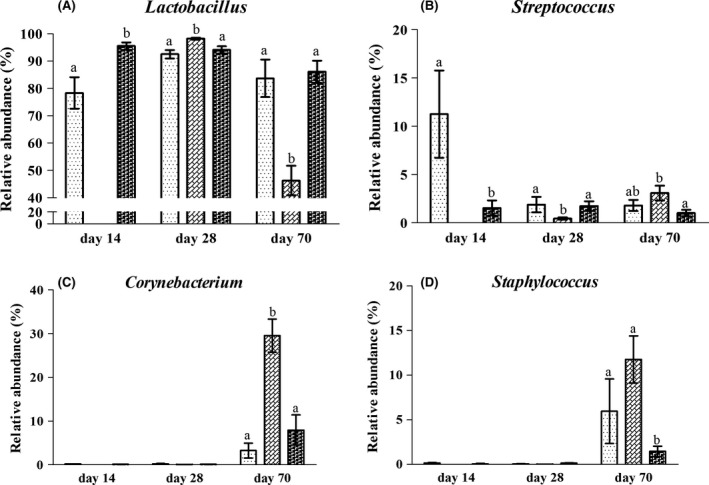
The most significantly altered abundance of bacteria in the jejunal content of rats at the genus level (A–D) in the L (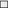), LN (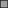) and N (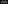) groups on day 14, day 28 and day 70. Values are presented as the mean ± SEM (*n *=* *6). Different letters among groups on the same day indicate a significant difference, *P *<* *0.05.

At the family and genus level, the top 20 dominant bacterial groups in the jejunum were selected to statistically investigate the differences in their relative abundance among the groups (Figs S5 and S6). On day 14, in response to the LPD, the abundance of *Lactobacillus* was lower in the jejunum of rats in the L group compared to the N group (Fig. 5A). However, the abundance of *Streptococcus*,* Rothia*,* Clostridium XI*, Lachnospiraceae, Aerococcaceae, *Clostridium sensu stricto* and *Turicibacter* was higher in the jejunum of rats in the L group. On day 28, the abundance of *Lactobacillus* and *Streptococcus* in the L group did not differ from the N group (Fig. 5B), while the abundance of Ruminococcaceae and *Clostridium sensu stricto* was enriched in the jejunum of rats in the L group compared to the N group. However, compared to the L and N groups, a significantly higher abundance of *Lactobacillus* and a remarkably lower abundance of *Streptococcus* were observed in the jejunum of rats in the LN group on day 28 in response to the diet switch from the LPD to the NPD. In addition, significantly lower abundance of *Olsenella* and Sphingomonadaceae was detected in the LN group compared to the N group. The abundance of *Clostridium sensu stricto* in the jejunum of rats in the LN group was lower than in the L and N groups on day 28. On day 70, the abundance of the top 20 bacterial groups in the L group did not significantly differ from that in the N group (Fig. 5C). However, a significantly lower abundance of *Lactobacillus* and a remarkably higher abundance of *Streptococcus* were observed in the jejunum of rats in the LN group compared to the N group. In addition, the abundance of *Corynebacterium*,* Staphylococcus, Jeotgalicoccus*,* Aerococcus, Facklamia*,* Pseudomonas*,* Novosphingobium*,* Gemella*, Alcaligenaceae, Bradyrhizobiaceae and *Allobaculum* was higher in the LN group. Moreover, the abundance of *Corynebacterium*,* Jeotgalicoccus* and *Facklamia* in the LN group was higher than in the L group on day 70. Overall, the abundance of *Lactobacillus*,* Streptococcus*,* Corynebacterium* and *Staphylococcus*, as the most dominant bacterial groups in the jejunal content, was altered most significantly during the experimental period (Fig. 6A–D). The abundance of *Corynebacterium* and *Staphylococcus* was abruptly higher in the jejunum of rats in the LN group on day 70 compared to the N group.

### Quantification of the total bacteria in the jejunal content by qPCR

**Figure 7:**
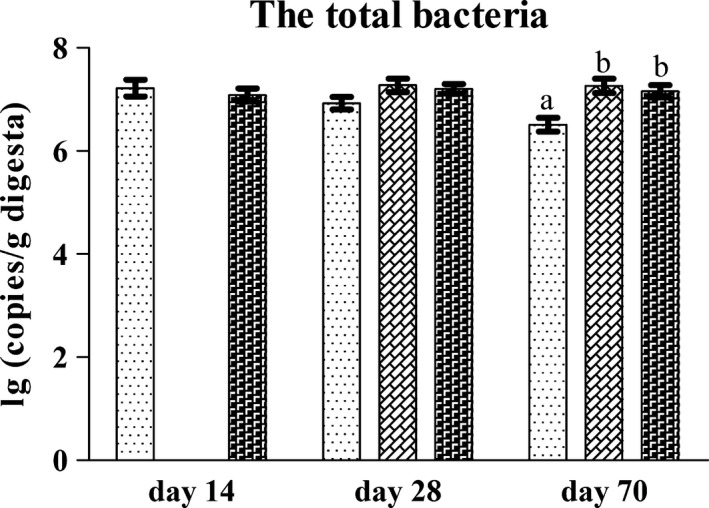
The gene copies of total bacteria in the jejunal content of rats in the he L (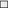), LN (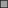) and N (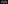) groups on day 14, day 28 and day 70. Values are presented as the mean ± SEM (*n *=* *6). Different letters among groups on the same day indicate a significant difference, *P *<* *0.05.

As MiSeq sequencing could not reveal the absolute number of bacteria in the jejunal content, the gene copies of total bacteria in the jejunal content were quantified by qPCR. The results (Fig. 7) showed that the number of total bacteria in the jejunal content of the rats in the L group did not differ from that in the N group on day 14. In addition, no difference was observed in the number of total bacteria in the jejunal content of rats in the L, LN and N groups on day 28. On day 70, the number of total bacteria in the jejunal content of the rats in the LN and N groups did not differ. However, the number of total bacteria in the jejunal content of the rats in the L group was significantly lower than that in the LN and N groups.

### Concentrations of SCFAs and ammonia‐N in the jejunal content

Table 2 shows the concentrations of detectable short chain fatty acids (SCFAs) and ammonia‐N in the jejunal content. The rats in the L group had a lower concentration of butyrate on day 14, lower concentrations of propionate and isobutyrate on day 28 and a decreased concentration of acetate on day 70 in the jejunum compared to the N group. However, the concentrations of SCFAs in the jejunum of rats in the LN group did not significantly differ from the N group on day 28 and day 70, despite a lower concentration of propionate on day 28. No difference was observed in the detected SCFAs between the L and LN groups on day 28 and day 70. The concentration of ammonia‐N decreased in the jejunum of rats in the L group on day 14 and day 28 compared to the N group. However, the concentration of ammonia‐N in the jejunum of rats in the LN group did not differ from that of the rats in the N group on day 28 and day 70. In addition, the ammonia‐N in the LN group was higher than that in the L group on day 28 and day 70.

**Table 2:** The concentration of SCFAs (μmol/g content) and ammonia‐N (mmol/g content) in the jejunal content

Item	L	LN	N	SEM	*P* value^a^
Day 14					
Acetate	9.16	–	9.10	0.55	0.96
Propionate	0.52	–	0.68	0.10	0.44
Isobutyrate	0.47	–	0.26	0.06	0.10
Butyrate	2.01^a^	–	0.77^b^	0.23	0.00
Ammonia‐N	416.83^a^		811.94^b^	73.22	0.01
Day 28					
Acetate	20.28	21.73	24.85	2.73	0.81
Propionate	1.45^a^	2.46^a^	5.71^b^	0.55	0.00
Isobutyrate	0.68^a^	0.81^ab^	1.34^b^	0.12	0.05
Butyrate	1.80	1.27	2.34	0.31	0.40
Ammonia‐N	377.67^a^	851.34^b^	769.15^b^	58.70	0.00
Day 70					
Acetate	4.74^a^	7.71^ab^	10.04^b^	0.89	0.04
Propionate	1.78	2.18	3.04	0.32	0.27
Isobutyrate	0.98	1.12	1.20	0.17	0.87
Butyrate	1.28	0.80	0.68	0.13	0.13
Ammonia‐N	476.46^a^	754.63^b^	610.05^ab^	39.68	0.01

Means in a same row with different superscripts indicate a significant difference (*n *=* *6, *P *<* *0.05).

a *P* value is the significance of independent samples t‐test or the significance of ANOVA.

### Correlation between mucosal genes and bacteria abundance

The abundance of *Lactobacillus* and the abundance of *Streptococcus* were both correlated with a different extent with the majority of gene expression tested in this study, as indicated by the *P* values and correlation coefficient (CC) (Table 3). In particular, the abundance of *Lactobacillus* was significantly positively correlated with the expression of *Apa*,* Cat‐1* and *Occludin* (CC > 0.5, *P *<* *0.05). However, the abundance of *Streptococcus* was significantly negatively correlated with the expression of *Apa* and *Occludin* (CC < −0.5, *P *<* *0.05). In addition, the abundance of *Corynebacterium* was significantly negatively correlated with the expression of *Glut‐2* (CC = −0.66, *P *<* *0.05) and *Occludin* (CC = −0.54, *P *<* *0.05) and significantly positively correlated with expression of *Tnf‐α* (CC = 0.81, *P *<* *0.05). Moreover, the abundance of *Staphylococcus* was significantly positively correlated with the expression of *Tnf‐α* (CC = 0.57, *P *<* *0.05).

**Table 3:** The correlation^a^ between the abundance of dominant bacterial groups and the gene expression

Genes	*Lactobacillus*		*Streptococcu*s		*Corynebacterium*		*Staphylococcu*s	
	CC	*P* value	CC	*P* value	CC	*P* value	CC	*P* value
*Apa*	0.52	0.00	−0.55	0.00	−0.14	0.57	−0.29	0.24
*Apn*	0.37	0.01	−0.30	0.04	−0.14	0.58	0.23	0.37
*Dpp‐4*	0.41	0.00	−0.46	0.00	−0.19	0.46	0.07	0.78
*Cat‐1*	0.51	0.00	−0.46	0.00	−0.26	0.30	−0.05	0.86
*Glut‐2*	0.49	0.00	−0.40	0.01	−0.66	0.00	−0.34	0.17
*Sglt‐1*	0.50	0.00	−0.45	0.00	−0.41	0.09	−0.29	0.25
*PepT‐1*	0.49	0.00	−0.41	0.00	−0.18	0.47	0.00	1.00
*Slc6a19*	0.47	0.00	−0.41	0.00	−0.34	0.17	−0.15	0.55
*Il‐10*	0.22	0.13	−0.11	0.44	−0.09	0.72	−0.13	0.61
*Tnf‐α*	0.17	0.25	−0.33	0.02	0.81	0.00	0.57	0.01
*Occludin*	0.54	0.00	−0.53	0.00	−0.54	0.02	−0.15	0.57
*Zo‐1*	0.18	0.22	−0.32	0.03	0.41	0.09	0.04	0.88

CC, correlation coefficient.

a The correlation was considered significant when the absolute value of correlation coefficient was >0.5 and statistically significant (*P *<* *0.05).

## Discussion

At the end of the experiment, the body weight of rats in the L group decreased compared to the N group, while the rats in the LN group experienced compensatory growth and showed undifferentiated body weight compared to rats in the N group. During the period of compensatory growth, the rats in the LN group had a higher feed conversion rate (from day 14 to day 28, LN vs. *N* = 0.61 vs. 0.41; from day 28 to day 70, LN vs. *N* = 0.31 vs. 0.24) compared to the rats in the N group, which was calculated as average daily gain/average feed intake (Zhu *et al*., 2017). According to previous studies, changes in circulating hormones and metabolic changes in multiple organs and tissues are believed to be reasons for or consequences of compensatory growth (Wilson and Osbourn, 1960; Ishida *et al*., 2011). Moreover, GH is a key hormone that regulates body weight and muscle growth (Dørup *et al*., 1992). Therefore, the elevated concentration of serum GH on day 28 might promote the growth of rats in the LN group. However, the main purpose of this study is to investigate the dynamic changes in morphology, gene expression and microbiome in the jejunum of compensatory‐growth rats due to the importance of jejunum to host.

The basic physiological function of jejunum is to digest and absorb nutrients and provide nutrients to the host. The higher VH and increased expression of genes related to digestion and absorption in the jejunum of rats in the LN group on day 28 indicated a higher capacity of nutrient digestion and absorption, which might contribute to the increased feed conversion rate and compensatory growth of rats. In addition, the higher expression of tight junction proteins suggested an improved barrier function to ensure the compensatory growth of rats in the LN group, as the intestinal epithelial barrier controls the equilibrium between tolerance and immunity to non‐self‐antigens (Fasano and Sheadonohue, 2005). Interestingly, the higher expression of *Cat‐1*,* Slc6a19*,* PepT‐1*,* Glut‐2*,* Sglt‐1* and *Zo‐1* was also found in the jejunum of rats in the L group on day 28 compared to the rats in the N group, suggesting an activated potency of gut function in response to protein restriction. These results agree with the findings of Zarling and Mobarhan (1987), who found that rats had normal or increased sucrase and lactase activities in the small intestine to support a normal amount of disaccharide hydrolysis in response to a balanced diet restriction. Moreover, previous studies demonstrated that enterocytes can directly utilize dietary nutrients during first‐pass metabolism (Stoll *et al*., 1998). Gene expression and the maintenance of gut morphology depend on the presence of luminal nutrients, particularly an adequate supply of amino acids (Howard *et al*., 2004; Wu, 2013). Therefore, increased nutrient availability due to the switch from LPD to NPD could further stimulate the potency of gene transcription in jejunal epithelial cells, resulting in higher expression of genes related to jejunal function in the LN group on day 28. Moreover, the decreased VH of rats in the L group on day 28 and day 70 and the inhibited gene expression related to jejunal function on day 14 laterally proved that a normal protein diet is essential for the gut function. Consistent with our findings, the previous studies showed that jejunal morphology and gene expression were disrupted when dietary protein was restricted (Solimano *et al*., 1967; Li *et al*., 2015).

However, improved parameters related to jejunal function were not consistently observed. The poor expression of absorption‐ and barrier‐associated genes and increased expression of *Tnf‐α* on day 70 in the jejunum of rats in the LN group suggested impaired gut absorptive and immune functions. This was possibly due to the epigenetic control of gene expression and the metabolic imprinting effects induced by early protein restriction, as revealed by previous studies (Park, 2005). In addition, De and Moura (2000) found that feeding postnatal rats a protein‐free diet would cause a metabolic imprinting that led to decreased insulin secretion and increased insulin sensitivity as an adaptive response. In agreement with this finding, we also detected decreased serum insulin of rats in the LN group on day 70, suggesting decreased insulin secretion or increased insulin sensitivity. Therefore, we speculated that the inhibited expression of genes in the jejunum on day 70 was due to the metabolic imprinting effects of early protein restriction as an adaptive response. However, to our knowledge, metabolic imprinting effects of early nutrition are rarely reported in post‐weaning animals, and more investigation is needed to verify the metabolic imprinting effects of protein restriction in post‐weaning animals. In addition, Tarryadkins *et al*. (2016) have shown that oxidative stress and inflammation usually accompany the compensatory growth of rats. Therefore, the elevated expression of *Tnf‐α* in the jejunum of rats in the LN group indicated chronic inflammation as a cost of the rapid growth rate.

Due to the significance of jejunal microbiota in affecting mucosal gene expression and metabolism as shown by Aidy *et al*. (2013), the jejunal microbiome was investigated and the correlation analysis between bacteria abundance and gene expression was conducted. The most significant changes were found in the abundance of *Lactobacillus*,* Streptococcus*,* Corynebacterium* and *Staphylococcus*. These results indicate that the compensatory growth of rats was accompanied by dramatic changes in the abundance of dominant bacterial groups in the jejunal content. According to previous studies, a major factor of changes in gut microbial composition is diet composition, particularly dietary protein (Daniel *et al*., 2013; Mu *et al*., 2016). Previous studies showed that *Lactobacillus* could actively degrade soybean proteins *in vitro* (Aguirre *et al*., 2014) and ingestion of soy products by rats would cause a slight increase in faecal lactobacilli (Bedani *et al*., 2010), while *Streptococcus spp*. were the major utilizers of available carbohydrates in the small intestinal lumen (Zoetendal *et al*., 2012). Therefore, the differences in the proportion of soybean and carbohydrates between LPD and NPD were associated with the changes in the abundance of *Lactobacillus* and *Streptococcus* on day 14 and day 28. In addition, the gut microbiome is also regulated by the host, particularly the gut immune system, which is an important contributor to “inside‐out” host control over microbiota composition (Hooper *et al*., 2012). The inhibited expression of genes related to barrier function and increased expression of *Tnf‐α* on day 70 in the jejunum of rats in the LN group indicated impaired gut innate immune function, allowing the proliferation of pathogenic bacteria. Therefore, the abundance of *Lactobacillus* decreased, while the abundance of opportunistic pathogenic bacteria, including *Corynebacterium*, and *Staphylococcus*, increased on day 70 in the LN group. This was validated by correlation analysis showing that both *Corynebacterium* and *Staphylococcus* were positively correlated with the expression of *Tnf‐α*. In addition, the correlation analysis revealed that the *Lactobacillus* and *Streptococcus*, as the most dominant bacterial groups, were significantly correlated with the gene expression in the jejunal mucosa. These results agreed with previous studies showing that *Lactobacillus* and *Streptococcus* can regulate gene transcripts in the small intestine, particularly the genes associated with gut barrier function (Walter, 2008; Distrutti *et al*., 2013; Van den Bogert *et al*., 2013; Tannock *et al*., 2014; Wang *et al*., 2014). However, the correlations between microbiota and gene expression related to nutrient digestion and absorption are rarely reported. Our results verified the hypothesis of Aidy *et al*. (2013) that microbiota in small intestine could affect gene expression in the mucosa.

In conclusion, the compensatory growth of protein‐restricted rats was accompanied by altered microbial composition in the jejunal content. The increased VH and expression of genes related to barrier function, nutrient digestion and absorption on day 28 might facilitate the compensatory growth of rats in the LN group. Moreover, the changes in the abundance of *Lactobacillus*,* Streptococcus*,* Corynebacterium* and *Staphylococcus* might be associated with the gene expression in the jejunal mucosa. However, the evaluation of jejunal function in this study was solely based on gene expressions and morphology. We were not able to investigate the changes in specific proteins due to the limited amount of samples. These issues will be investigated in our current research on piglets. In addition, future studies should apply transcriptomics and proteomics to fully reveal the changes in gene transcription and protein expression of jejunal mucosa in compensatory‐growth rats.

## Experimental procedures

### Animal trial

Forty‐eight Sprague‐Dawley male rats with an initial average body weight of 44.7 ± 1.51 g, weaned at 21 days of age, were used in the study. Eighteen rats were randomly selected to be fed a NPD as a control group (N group), with three rats housed per cage. The remaining 30 rats (L group) were randomly allocated into six cages and fed with LPD until experimental day 14, with five rats per cage. Diets were formulated according to the AIN‐93G formula designated for the growth, pregnancy and lactational phases of rodents (Reeves *et al*., 1993) (Table S1). On day 14, six rats from the N group and the other six rats from the L group were sacrificed for sampling. On the same day, the rats from the L group were mixed and randomly reassigned to an L group (rats were continuously fed LPD) and an LN group (rats were fed NPD). To exclude the mixing effect in the L and LN groups, the rats in the N group were also mixed and reassigned to six cages, with two rats in each cage. On days 28 and 70, six rats per group were sacrificed for sampling. During the entire experimental period, rats were raised at constant temperature (25°C) and humidity (70%) on a 12‐h light/dark cycle. Water and feed were fed *ad libitum* throughout the experiment.

### Sampling

Blood was sampled before the rats were sacrificed and was stored at −80°C until analysis. The rats were killed by decapitation. The abdomen was opened, and the entire gastrointestinal tract was removed. Jejunal content was collected in a sterile tube and immediately stored at −20°C for later bacterial DNA and metabolite analyses. Jejunal mucosa was collected in a sterile tube by scraping with slides and immediately stored in liquid nitrogen for later RNA extraction. In addition, 2 cm of proximal jejunal tissues were fixed in 4% paraformaldehyde solution for morphology analysis.

### Jejunal morphology

After fixing for 24 h, jejunal samples were dehydrated using graded concentrations of ethanol (70–100%) and cleared with xylene. The samples were embedded in paraffin. Cross sections of the tissues were cut at a thickness of 5 μm with a microtome (American Optical Co., Scientific Instrument Division, Buffalo, NY, USA) and stained with haematoxylin and eosin (Chen *et al*., 2014; Pi *et al*., 2014). The sections were visualized using Computer‐Aided Video Microscopy (DXM1200C; Nikon Inc, New York, USA). Villous height (VH) and crypt depth (CD) were measured and analysed using NIS‐Elements BR software (version 2.20; Nikon). To avoid subjectivity, the measurement was performed by a person who was not aware of the experimental details.

### Determination of serum hormones and microbial metabolites in jejunal content

Serum hormones, including glucagon, insulin, growth hormone (GH) and leptin, were detected by ELISA kits (Angle Gene, Nanjing, China) according to the recommended procedures. The ammonia‐N in the jejunal content was determined using the method described by Weatherburn (Weatherburn, 1967). The concentrations of SCFAs in the jejunal content were determined by gas chromatography as previously described (Mao *et al*., 2008; Zhu *et al*., 2017). The temperatures of the injector, column and detector were 110, 135 and 180°C respectively. All assays were performed in triplicate to acquire the average values. The intra‐assay (within plate) coefficient of variation (CV) for the ELISA was <9.0%, and the interassay (between plate) CV was <15%.

### Gene expression analysis and quantification of total bacteria by qPCR

The total RNA of jejunal mucosa was isolated using RNAiso Plus Reagent (TaKaRa Biotechnology, Dalian, China) according to the recommended protocol. The quality and quantity of RNA were assessed by agarose gel electrophoresis and a spectrophotometer (NanoDrop, ND‐100; Saveen Werner, Limhamn, Sweden). The samples were adjusted to the same concentration of RNA and reverse‐transcribed to complementary DNA using a PrimeScript RT reagent Kit with a gDNA eraser (TaKaRa Biotechnology) according to the recommended procedures. Quantitative PCR was performed on an Applied Biosystems 7300 Real‐Time PCR system (Foster City, CA, USA) using a SYBR Premix Ex Tag™ (Tli RnaseH Plus) qPCR kit (TaKaRa, Dalian, China) according to the manufacturer's guidelines. The reactions were run in duplicate for target genes and in triplicate for the housekeeping gene. The cycling conditions were 95°C × 30 s, followed by 40 cycles of 95°C × 5 s and 60°C × 34 s. The primers used are listed in Table S2. The gene expression cycle threshold (*C*_t_) values were recorded, and β‐actin was used as the housekeeping gene. The *C*_t_ value for the target genes of each sample was corrected by subtracting the C_t_ value of the *â*‐actin from the *C*_t_ value of the corresponding gene (Δ*C*_t_). The samples collected from the N group on each sampling day were used as the reference samples. The Δ*C*_t_ value of all samples was subtracted from the average Δ*C*_t_ of the reference samples (ΔΔ*C*_t_). The fold change in the expression of target genes was calculated using the $2^{-\Delta \Delta C_{t}}$ method (Livak and Schmittgen, 2001). The gene copies of total bacteria in the jejunal content were quantified using the standard curve method with qPCR (Rinttilä *et al*., 2004). Primer pairs for the total bacteria were 5′‐ACTCCTACGGGAGGCAGCAG‐3′ and 5′‐ATTACCGCGGCTGCTGG‐3′.

### Illumina MiSeq sequencing and data processing

Bacterial DNA was extracted using a bead‐beating method and phenol–chloroform extraction (Zoetendal *et al*., 1998). After DNA extraction and quantification, the forward primer 319F (ACTCCTACGGGAGGCAGCAG) and reverse primer 806R (GGACTACHVGGGTWTCTAAT) were used to amplify the V3–V4 regions of the bacterial 16S rRNA gene as previously described (Zhu *et al*., 2017). The amplicons, which were extracted from 2% agarose gels, were purified using an Axyprep DNA Gel Extraction Kit (Axygen Biosciences, Union City, CA, USA) according to the recommended instructions and quantified using Quantifluor™‐ST (Promega, Madison, WI, USA). The purified amplicons were pair‐end sequenced on an Illumina MiSeq platform according to a standard protocol (Caporaso *et al*., 2012).

The raw FASTQ files were de‐multiplexed and quality‐filtered using QIIME (version 1.70, Flagstaff, AZ, USA) using the standard criteria described previously (Mao *et al*., 2015). The OTUs were clustered with a 97% similarity cut‐off using UPARSE (version 7.1 http://drive5.com/uparse/), and the chimeric sequences were identified and removed using UCHIME (Edgar, 2010). The most abundant sequences within each OTU were designated representative sequences and classified using the Ribosomal Database Project (RDP) classifier with a standard minimum support threshold of 80% (Wang *et al*., 2007). The microbial community diversity was estimated using the observed species, Simpson and Shannon indices. The differences in sequence abundance between groups were evaluated with nonparametric tests.

### Statistical analysis

Data were analysed using SPSS 16.0 (IBM, New York, NY, USA) and expressed as the means ± SEM. Both parametric (t‐test and ANOVA) and nonparametric methods (Kruskal–Wallis test and Mann–Whitney *U*‐test) were used to assess the differences between treatments. The normality of the distribution of variables was tested by the Shapiro–Wilk test. The *t*‐test and Mann–Whitney *U*‐test were used to analyse data with a normal or non‐normal distribution on day 14 respectively. A one‐way ANOVA with LSD post hoc comparison procedure was used to analyse the data with a normal distribution and a non‐significant test of homogeneity of variance on day 28 and day 70. In addition, data with a non‐normal distribution or a significant test of homogeneity of variance on day 28 and day 70 were analysed by the Kruskal–Wallis test followed by the Mann–Whitney *U*‐test (Etxeberria *et al*., 2015). The Mann–Whitney *U*‐test was used to compare the differences between two groups. In the case of multiple comparisons of the microbial data, *P* values were adjusted with a false discovery rate (FDR) analysis (Benjamini and Hochberg, 1995), limiting the overall false discovery rate to 5% (*q *<* *0.05). Differences were considered significant when *P *<* *0.05. The correlation between mucosal gene expression and bacteria abundance was analysed using Spearman's correlation analysis in SPSS 16.0 (IBM, New York, USA). The correlation was considered significant when the absolute value of the correlation coefficient was >0.5 and statistically significant (*P *<* *0.05).

## Conflict of interest

None declared.

## Supporting information


**Fig. S1.** Species accumulation curve.
**Fig. S2.** Rarefaction curves of observed species, Shannon index and Simpson index.
**Fig. S3.** The bacterial diversity as indicated by the Shannon index and Simpson index in the jejunal content of rats in each group on day 14, day 28 and day 70.
**Fig. S4.** The relative abundance of predominant bacteria in the jejunal content of each rat in every group at the phylum level on day 14 (A), day 28 (B), and day 70 (C).
**Fig. S5.** The relative abundance of predominant bacteria in the jejunal content of each rat in every group at the family level on day 14 (A), day 28 (B), and day 70 (C), respectively.
**Fig. S6.** The relative abundance of predominant bacteria in the jejunal content of each rat in every group at the genus level on day 14 (A), day 28 (B), and day 70 (C), respectively.
**Table S1.** Diet formula used in this study.
**Table S2.** Primers used in this study.
**Table S3.** The average clean data acquired during sequencing.
