# Supplementary material for: Dynamic changes in morphology, gene expression and microbiome in the jejunum of compensatory‐growth rats induced by protein restriction
Source: Microb Biotechnol. 2018 Apr 6;11(4):734–46. doi: 10.1111/1751-7915.13266 (PMC6033190; doi:10.1111/1751-7915.13266)
Supplement: Supplementary file 1 — Fig. S1. Species accumulation curve. Fig. S2. Rarefaction curves of observed species, Shannon index and Simpson index. Fig. S3. The bacterial diversity as indicated by the Shannon index and Simpson index in the jejunal content of rats in each group on day 14, day 28 and day 70. Fig. S4. The relative abundance of predominant bacteria in the jejunal content of each rat in every group at the phylum level on day 14 (A), day 28 (B), and day 70 (C). Fig. S5. The relative abundance of predominant bacteria in the jejunal content of each rat in every group at the family level on day 14 (A), day 28 (B), and day 70 (C), respectively. Fig. S6. The relative abundance of predominant bacteria in the jejunal content of each rat in every group at the genus level on day 14 (A), day 28 (B), and day 70 (C), respectively. Table S1. Diet formula used in this study. Table S2. Primers used in this study. Table S3. The average clean data acquired during sequencing. [file MBT2-11-734-s001.docx]

**Supplemental** **Table 1.** Diet**^1^** formula used in this study.

| Ingredients（g/1000g） | NPD | LPD |
| --- | --- | --- |
| corn | 491.50 | 611.50 |
| wheat | 44.50 | 150.00 |
| cornstarch | 59.10 | 50.00 |
| soybean meal | 279.90 | 42.50 |
| fish meal | 20.00 | 20.00 |
| soybean oil | 50.00 | 50.00 |
| L-lysine | 4.50 | 11.30 |
| DL-methionine | 3.70 | 4.80 |
| L-threonine | 1.00 | 3.10 |
| limestone | 26.10 | 30.00 |
| calcium hydrogen phosphate | 14.00 | 21.50 |
| salt | 2.00 | 2.00 |
| vitamin mix**^2^** | 1.10 | 1.00 |
| mineral mix**^3^** | 2.50 | 2.40 |
| total | 1000.00 | 1000.00 |
| Nutritional value |  |  |
| digestible energy (Mcal/kg) | 3.40 | 3.40 |
| crude protein % | 19.34 | 12.00 |
| Lys % | 1.37 | 1.30 |
| Met % | 0.68 | 0.69 |
| (Met + Cys) % | 0.96 | 0.83 |
| Thr % | 0.84 | 0.70 |
| apparent P % | 0.40 | 0.51 |
| total P % | 0.61 | 0.68 |
| Ca % | 1.42 | 1.68 |
| DM % | 89.10 | 89.33 |

**^1^**formulated according to the AIN-93G;

**^2^**formulated according to the Vitamin Mix V10001;

**^3^**formulated according to the Mineral Mix S10026.

**Supplemental** **Table 2.** Primers used in this study.

| Gene name | Accession number | Primer sequences | Reference |
| --- | --- | --- | --- |
| *β-actin* | NM_031144.3 | sense-CACGATGGAGGGGCCGGACTCATC | (1) |
|  |  | antisense-TAAAGACCTCTATGCCAACACAGT |  |
| *Apa* | AF214568 | sense-GAGGCAGCGTGGAACTATACTC | (2) |
|  |  | antisense-AGGAGATGTAGCGGATGACTGT |  |
| *Apn* | NM_031012 | sense-GAAGGTGGCAACAAGAAAGTG | (2) |
|  |  | antisense- CGATATAGGCCAGCAGGTATGT |  |
| *Dpp-4* | NM_012789 | sense- AGGAGCATATTGAAACGAGTGC | (2) |
|  |  | antisense-ACAGATAATCGCTGGTCAGAGC |  |
| *Sglt1* | NM_013033 | sense-TTCTGTCCCTGCTCCTCTACAT | designed in this study |
|  |  | antisense-TCCTACTTCTCGGAAAGCAAAC |  |
| *Glut 2* | NM_012879 | sense-CATTGCTGGAAGAAGCGTATC |  |
|  |  | antisense-AGCAGATAGGCCAAGTAGGATG |  |
| *Cat-1* | NM_013111 | sense-AGCAGATAGGCCAAGTAGGATG |  |
|  |  | antisense-ATCACGAACCACAAGCTGA |  |
| *Slc6a19* | NM_001039722 | sense-GGTTCAACAAGGACATCGAGTT |  |
|  |  | antisense-GTCAGGGTCCCATACGCTATAC |  |
| *PepT-1* | NM_001079838 | sense-GAGTATGTTCTGTTCGCCTCCT |  |
|  |  | antisense-AGACAGGTTCCAACGAGGAATA |  |
| *Il-10* | NM_012854.2 | sense-GGAGTGAAGACCAGCAAAGG |  |
|  |  | antisense-GGCAACCCAAGTAACCCTTA |  |
| *Tnf-α* | NM_012675.3 | sense-GGGCTCAGAATTTCCAACAA |  |
|  |  | antisense-ATCCACTCAGGCATCGACAT |  |
| *Occludin* | NM_001106266.1 | sense-CCTTACAGGCCGGATGAAT |  |
|  |  | antisense-GCTCTGTCCCAAGCAAGTGT |  |
| *Zo-1* | NM_031329.2 | sense-CCTTTCGCCTGAAACAAACC |  |
|  |  | antisense-CTACATGCGACGGCAATGAC |  |

**Supplemental Table 3.** The average clean data acquired during sequencing.

|  | Groups | Raw data | Valid data | Valid% | Q20% | Q30% | GC% |
| --- | --- | --- | --- | --- | --- | --- | --- |
| day 14 | L | 57526.00 | 56863.70 | 98.85 | 96.18 | 88.28 | 52.38 |
|  | N | 62692.20 | 61979.70 | 98.86 | 96.33 | 88.63 | 52.56 |
| day 28 | L | 39741.80 | 39280.70 | 98.85 | 96.16 | 88.29 | 52.05 |
|  | LN | 44976.00 | 44476.30 | 98.89 | 96.19 | 88.38 | 52.76 |
|  | N | 51462.70 | 50702.00 | 98.45 | 96.01 | 87.89 | 53.21 |
| day 70 | L | 46001.60 | 45461.20 | 98.82 | 96.29 | 88.59 | 53.32 |
|  | LN | 57300.30 | 56389.00 | 98.49 | 96.19 | 88.36 | 53.54 |
|  | N | 49733.50 | 49048.00 | 98.64 | 96.11 | 88.12 | 53.31 |

The number of average raw sequences detected in a group was at least 39741 reads, with 39280 valid sequences. The indexes of quality control Q20 and Q30 were sufficient to ensure the accuracy of sequencing.


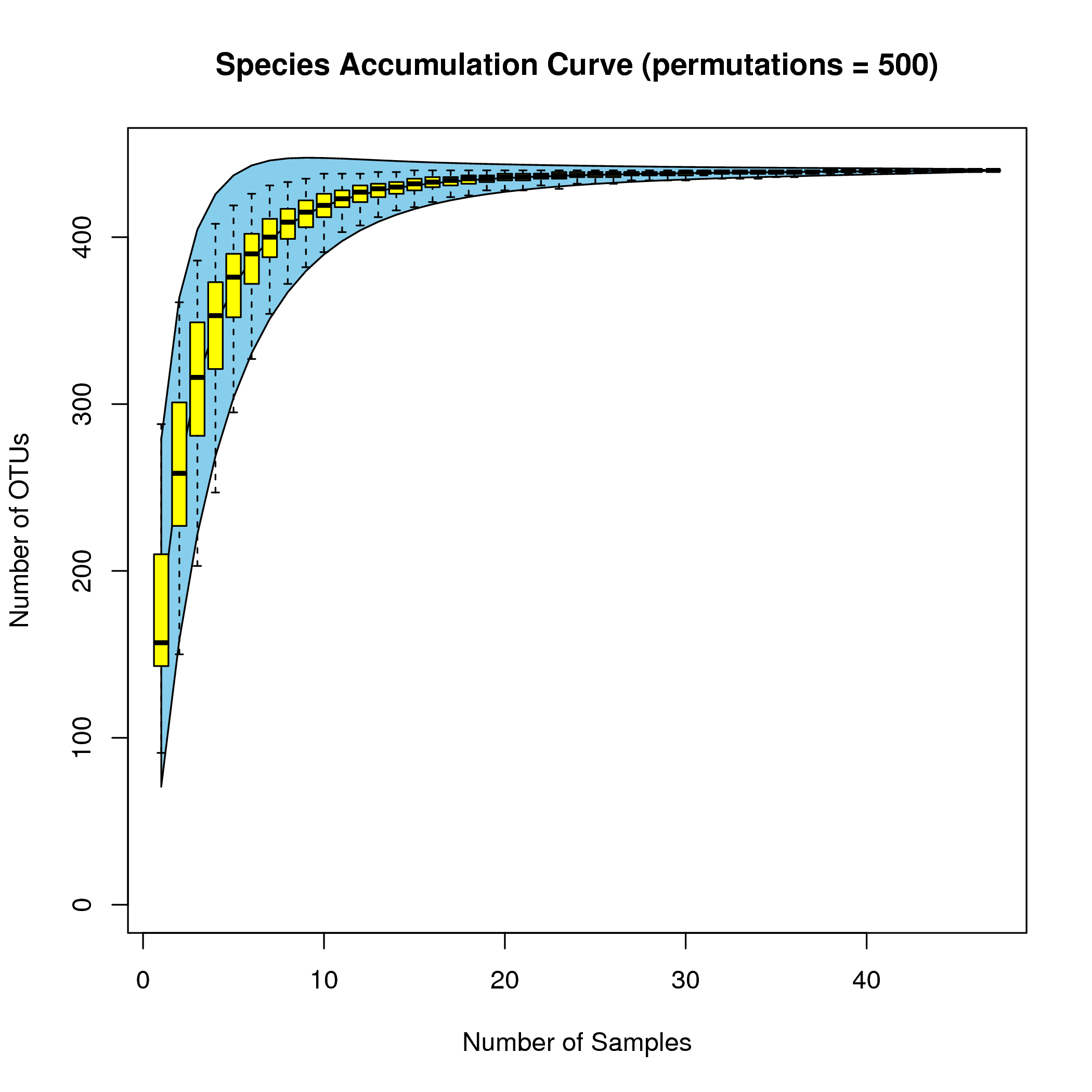


**Supplemental** **Figure 1.** Species accumulation curve. The overall number of OTUs detected was 440, based on a 97 % sequence similarity between reads.


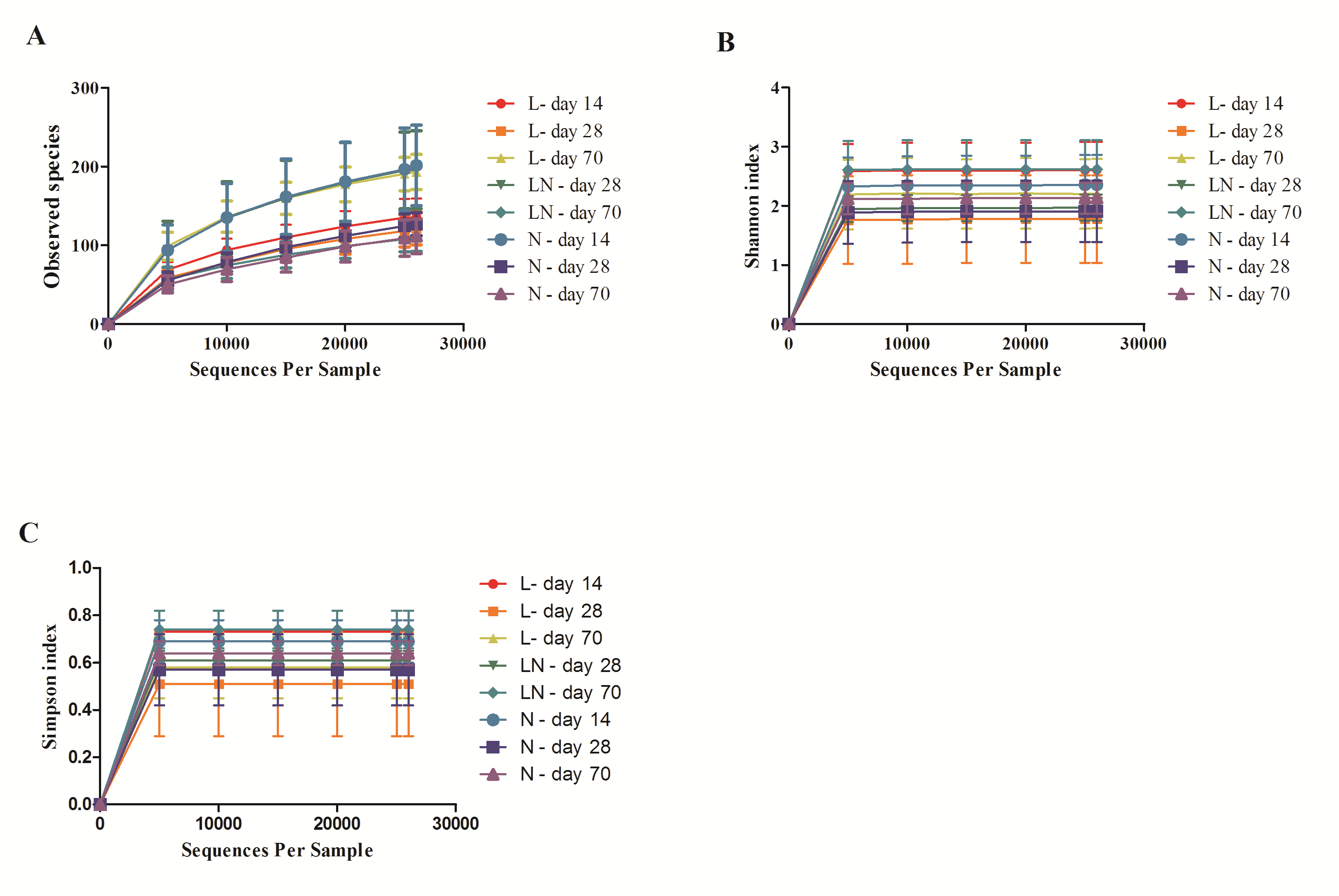


**Supplemental** **Figure 2.** Rarefaction curves of observed species, Shannon index and Simpson index. Results indicate that the sampling in this study was sufficient to evaluate the bacterial community profile.


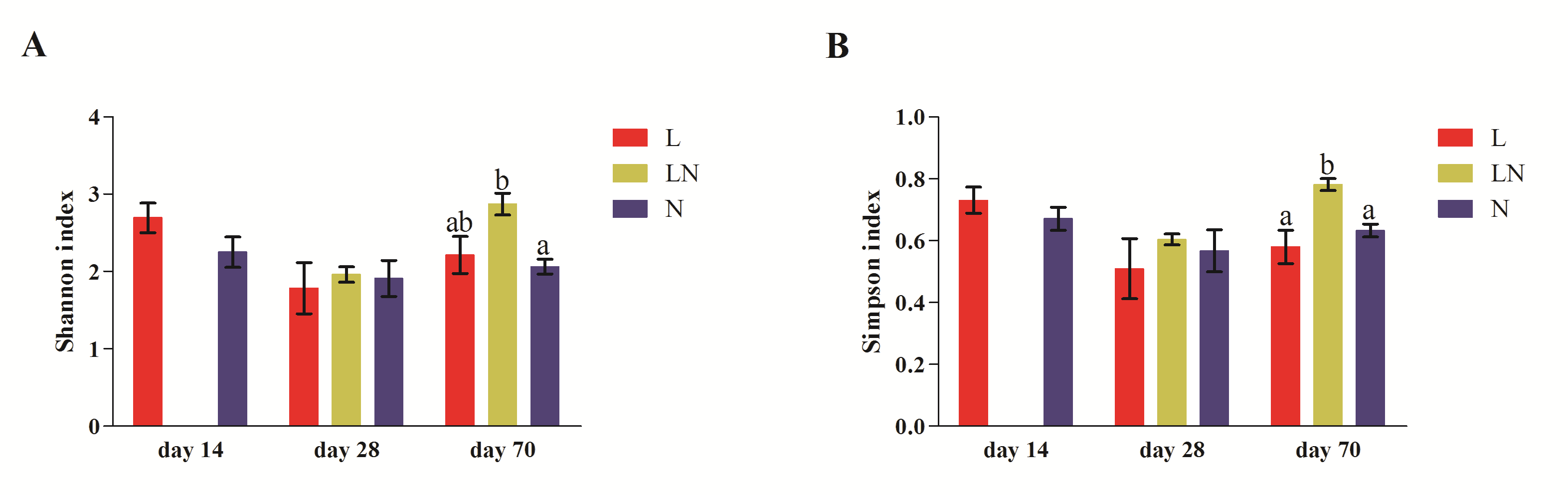


**Supplemental** **Figure 3.** The bacterial diversity as indicated by the Shannon index and Simpson index in the jejunal content of rats in each group on day 14, day 28 and day 70. Values are presented as the mean ± SEM (*n* =6). Different letters among groups on the same day indicate a significant difference, *p* < 0.05.

**C**


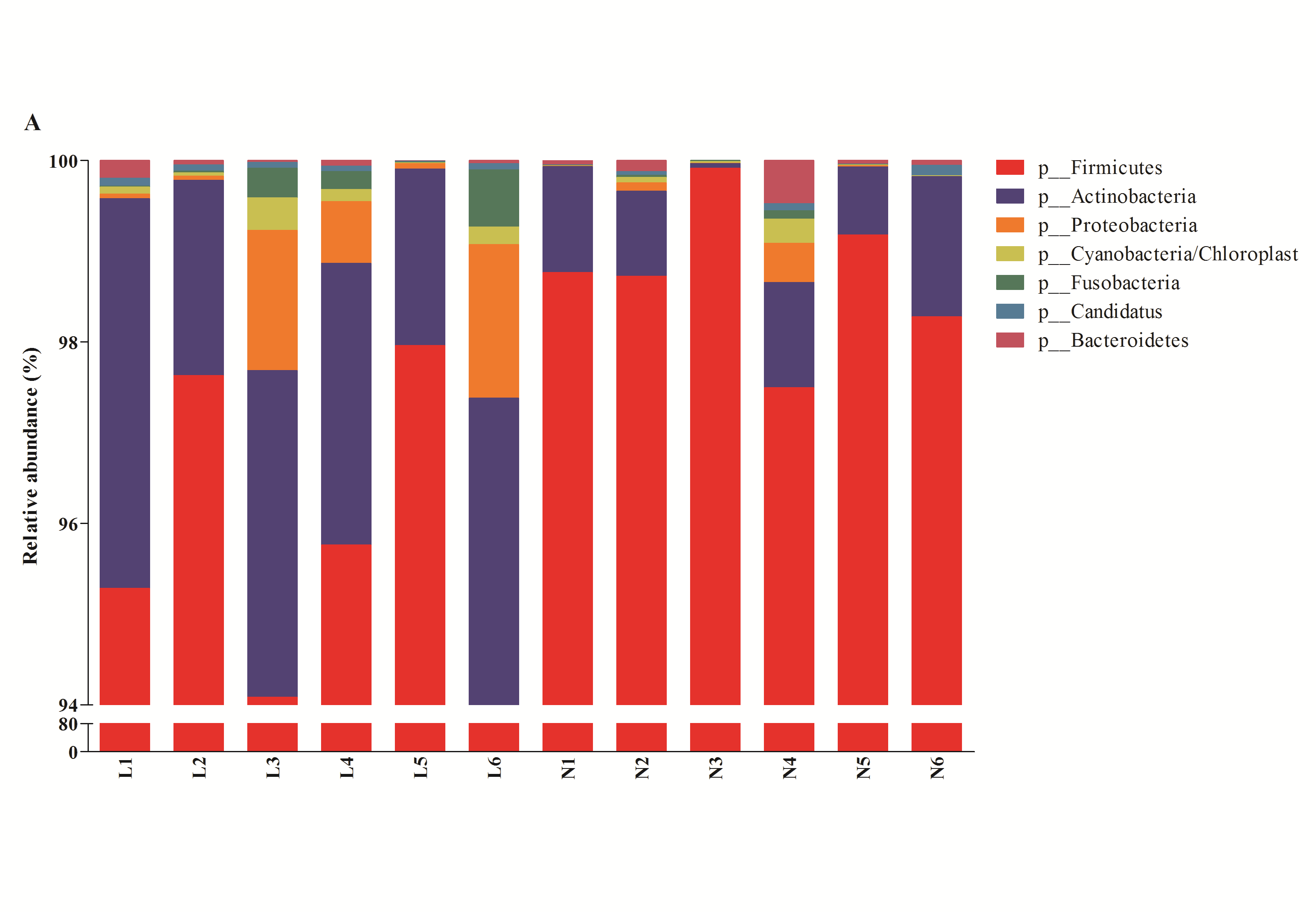

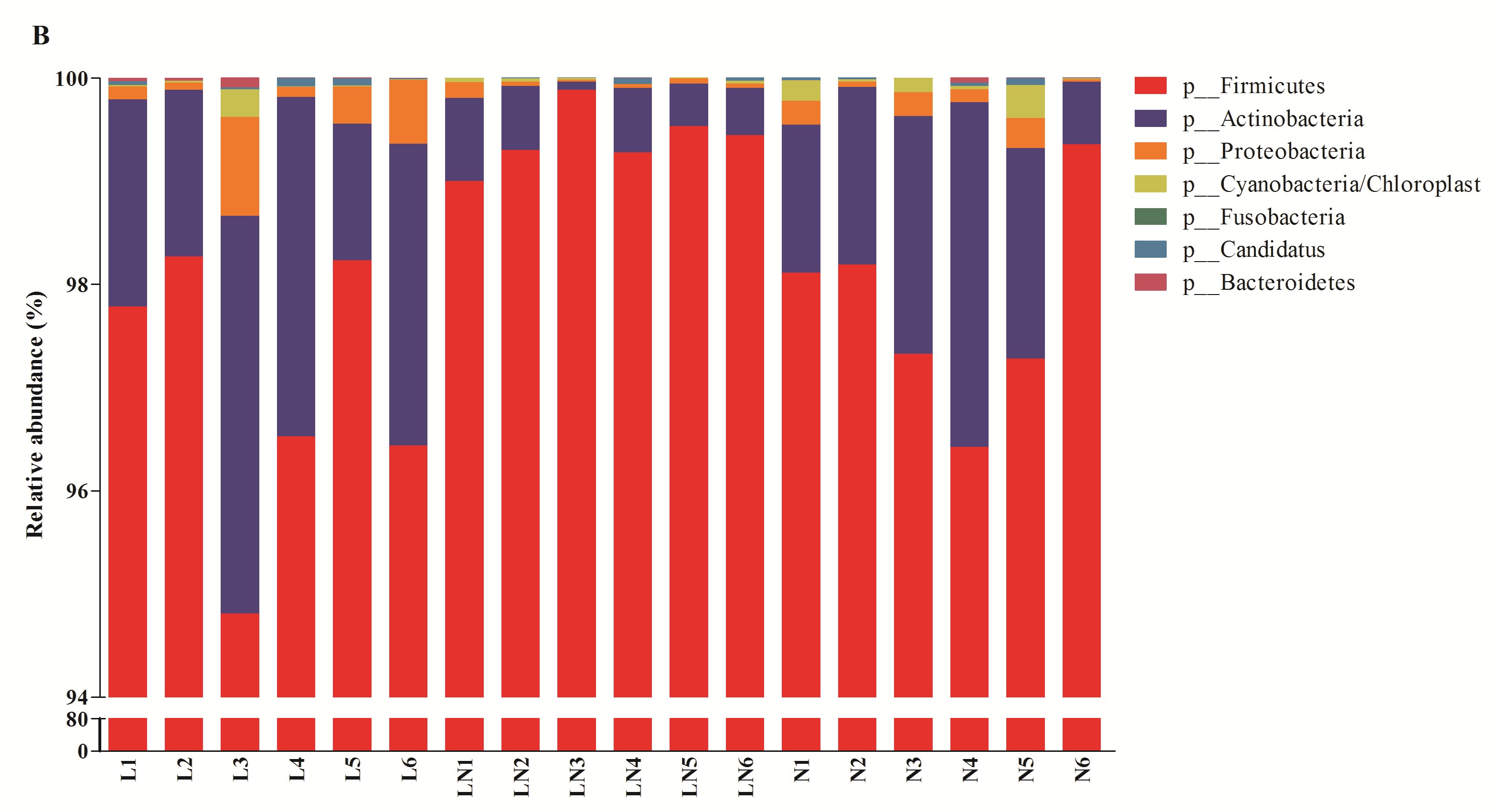

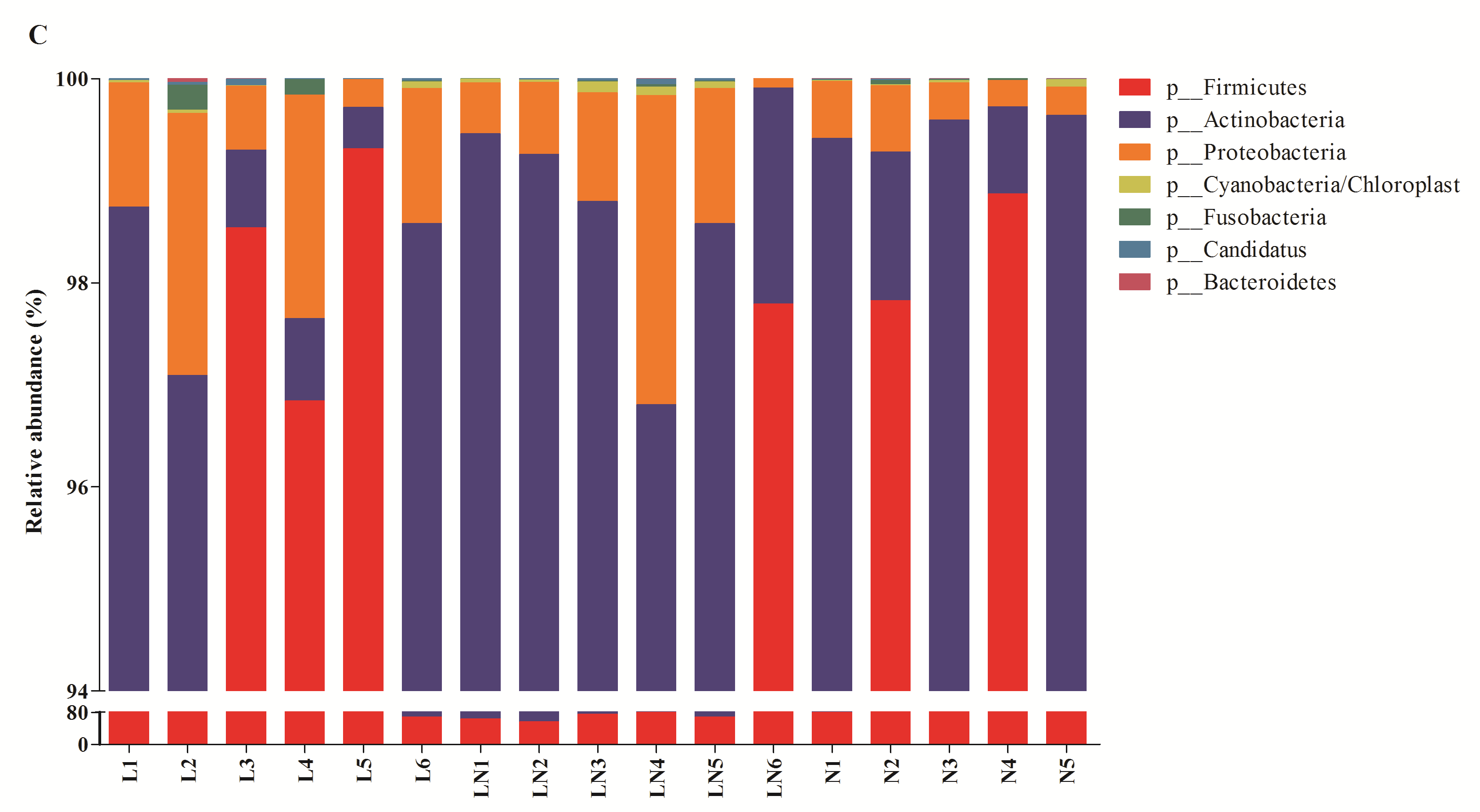


**Supplemental** **Figure 4.** The relative abundance of predominant bacteria in the jejunal content of each rat in every group at the phylum level on day 14 (**A**), day 28 (**B**), and day 70 (**C**).


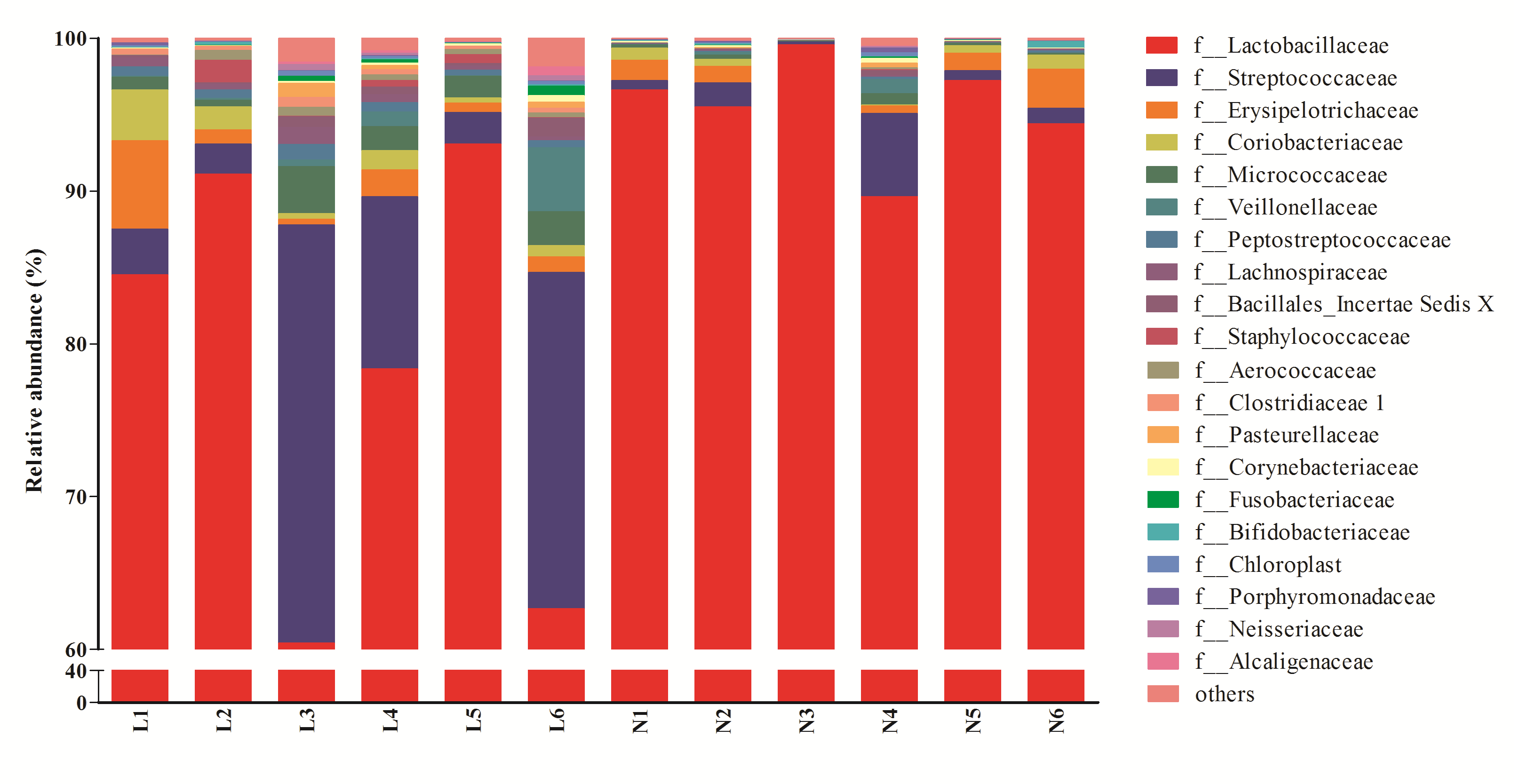


**C**

**B**

**A**


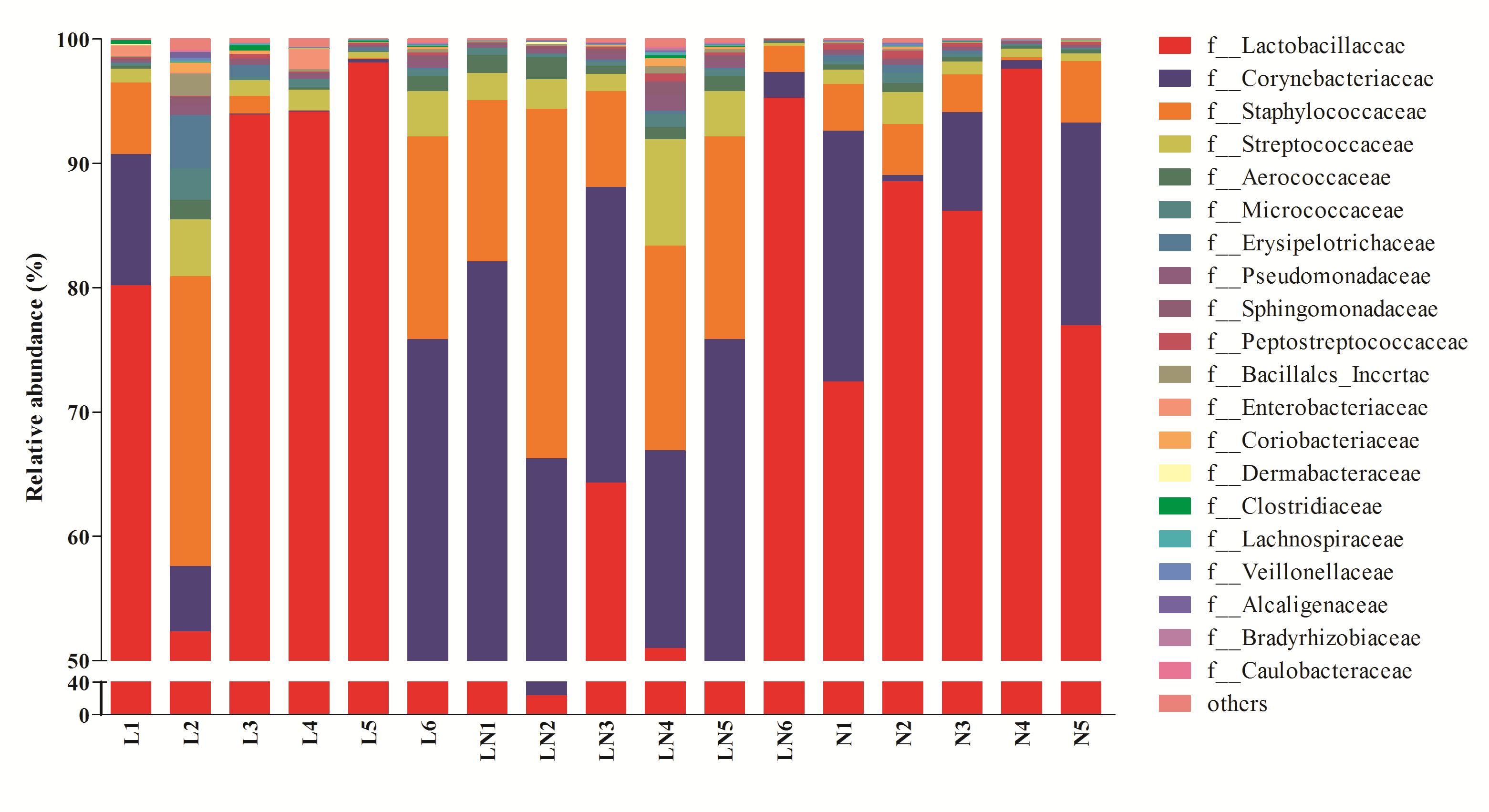

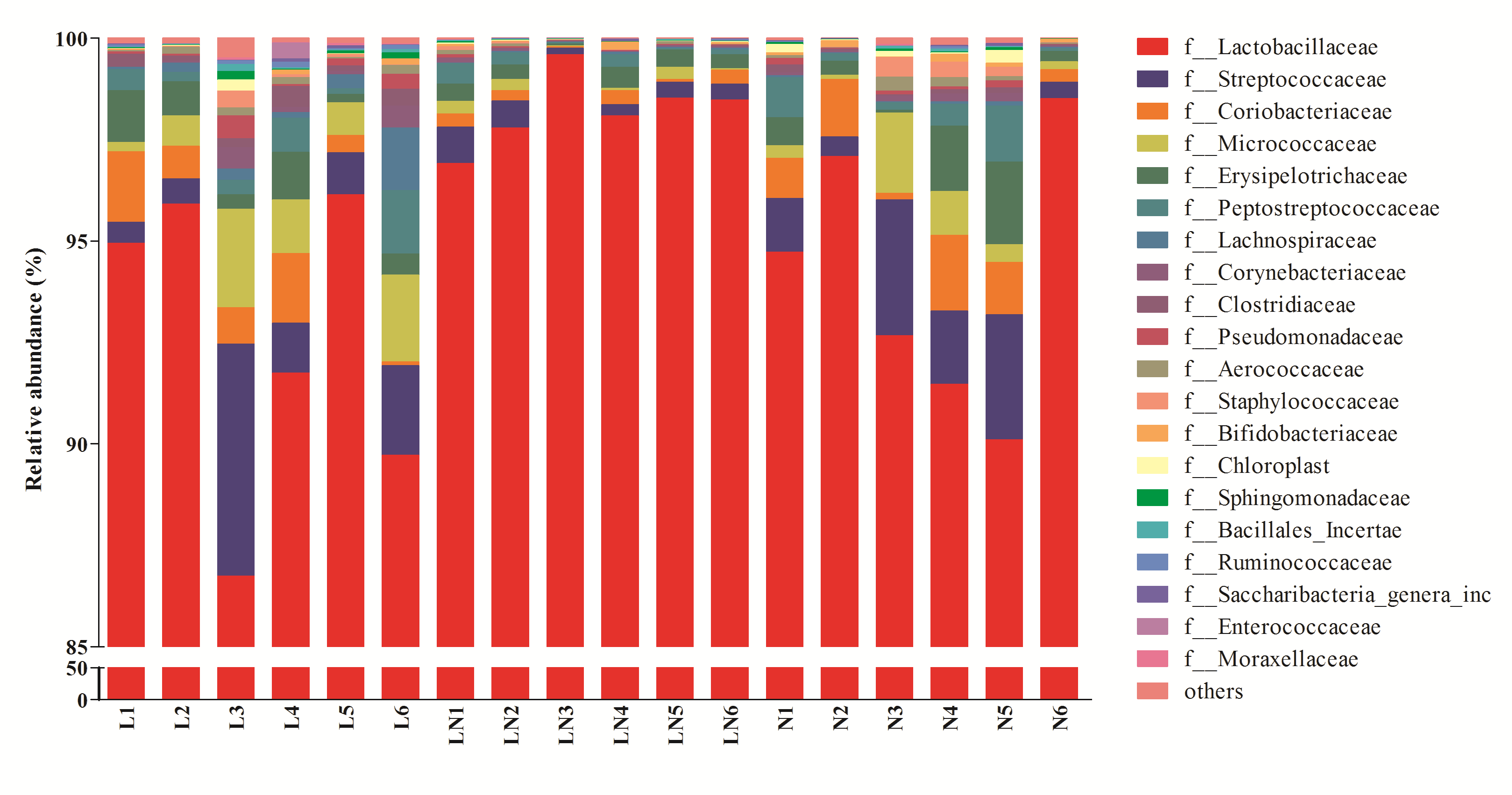


**Supplemental** **Figure 5.** The relative abundance of predominant bacteria in the jejunal content of each rat in every group at the family level on day 14 (**A**), day 28 (**B**), and day 70 (**C**), respectively.

**A**


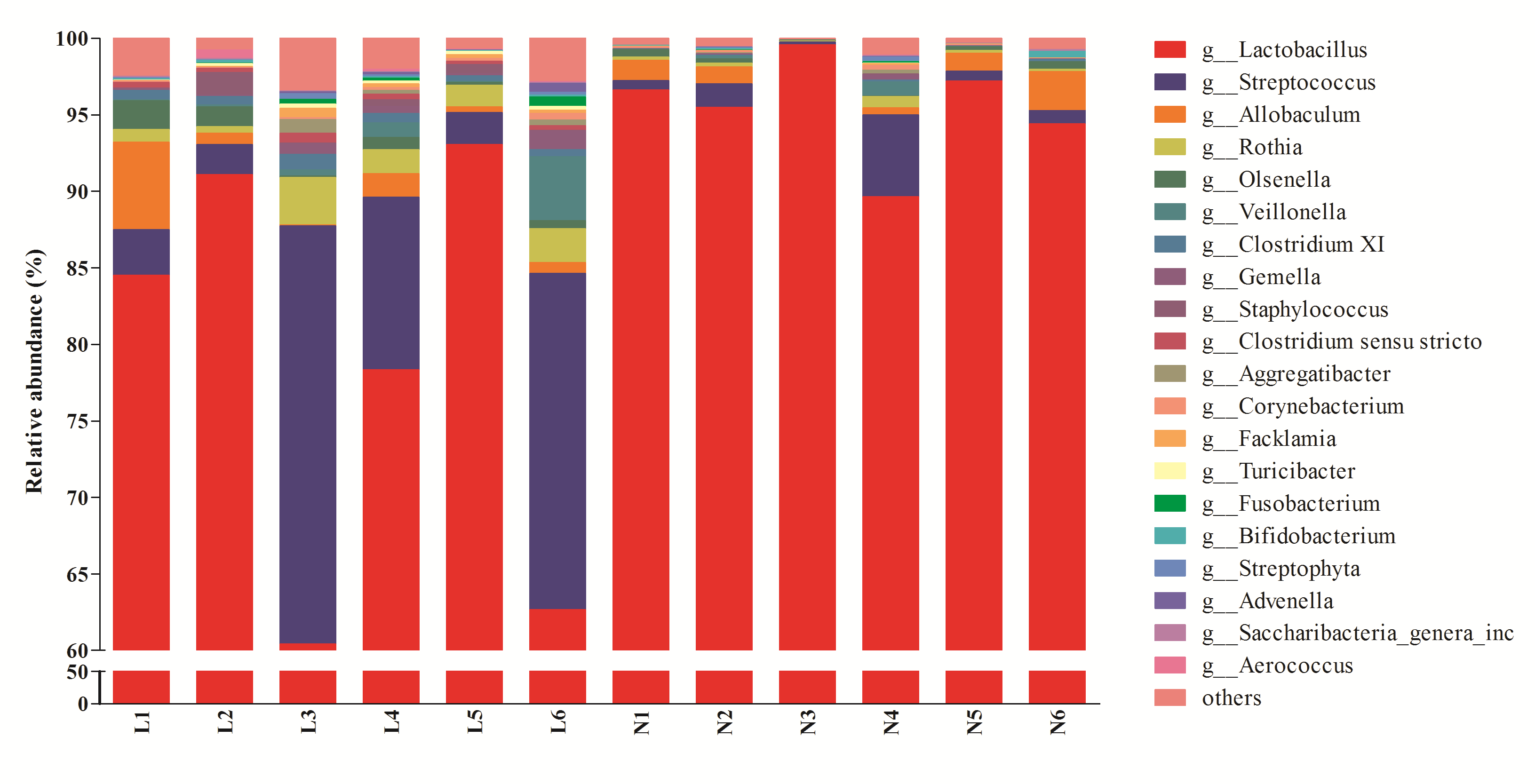


**B**


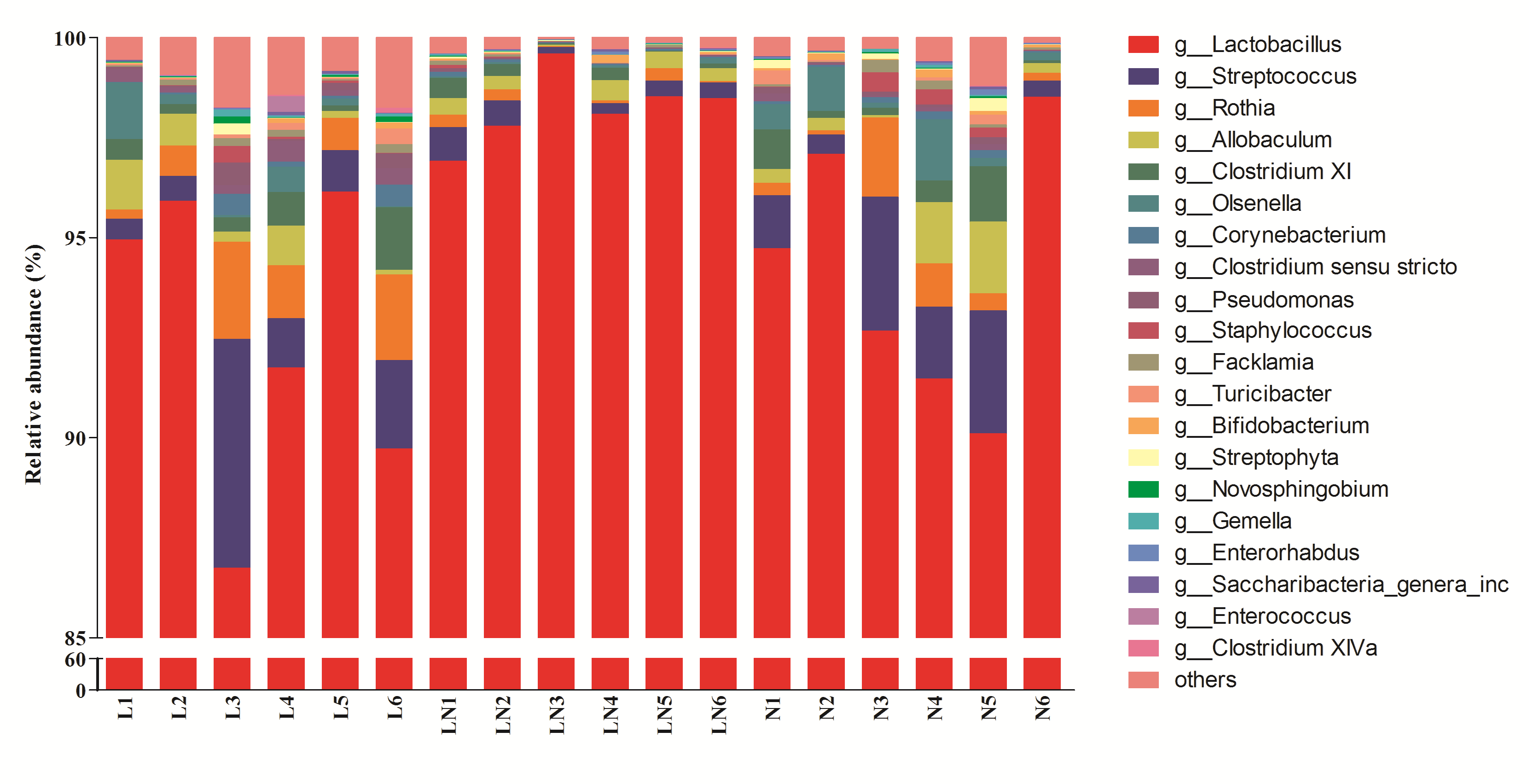


**C**


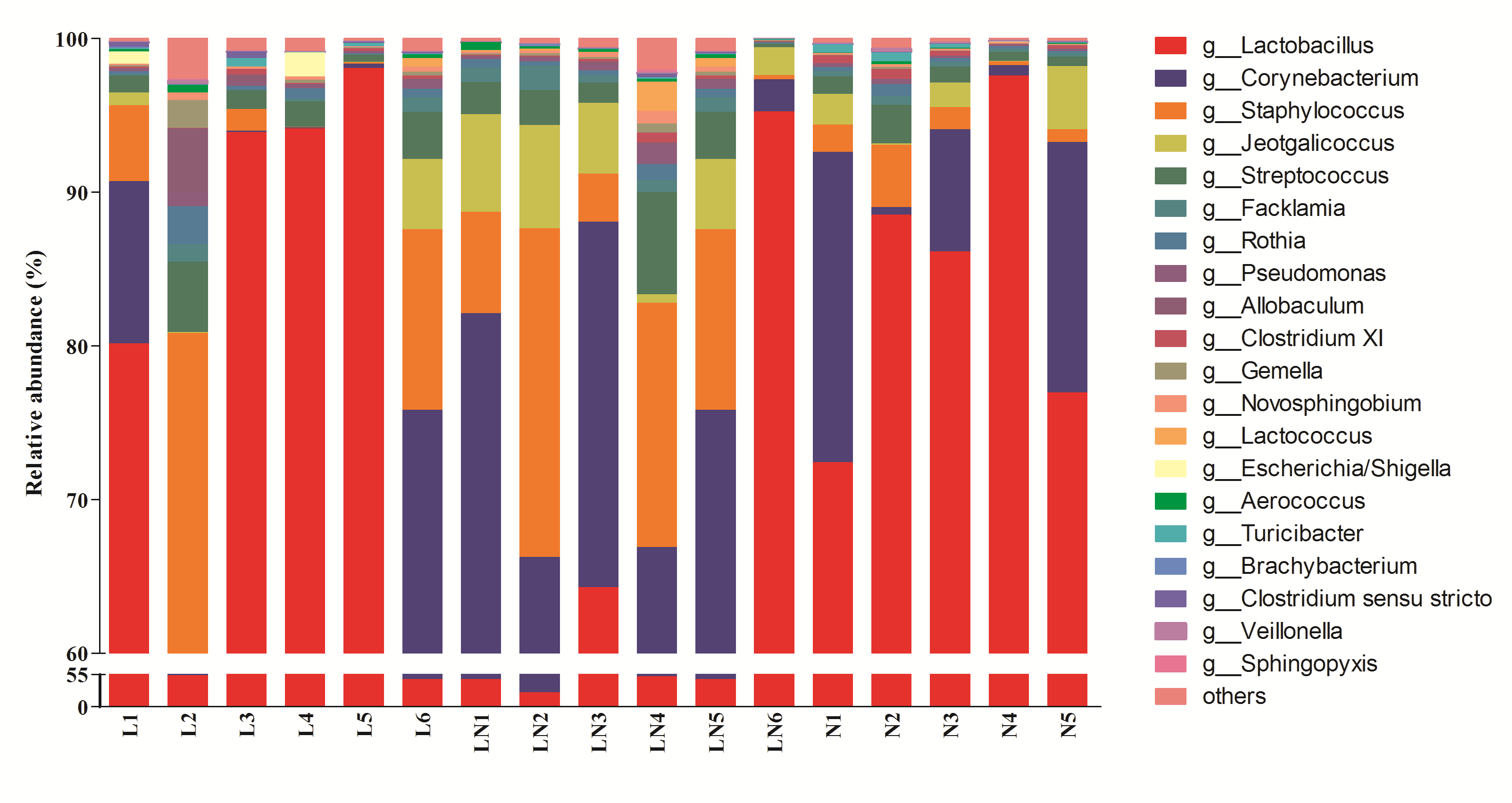


**Supplemental** **Figure 6.** The relative abundance of predominant bacteria in the jejunal content of each rat in every group at the genus level on day 14 (**A**), day 28 (**B**), and day 70 (**C**), respectively.

**Supplemental References**

1. Lin, J., Tsuboi, Y., Pan, W., Giebink, GS., Adams, GL., Kim, Y. (2002) Analysis by cDNA microarrays of altered gene expression in middle ears of rats following pneumococcal infection. *Int J Pediatr Otorhinolaryngol* **65**:203-211.
2. Zhou, J-Y., Zhou, S-W.. (2011) Protective effect of berberine on antioxidant enzymes and positive transcription elongation factor b expression in diabetic rat liver. *Fitoterapia* **82**:184-189.
